# Supplementary material for: Seminoma and Embryonal Carcinoma Footprints Identified by Analysis of Integrated Genome-Wide Epigenetic and Expression Profiles of Germ Cell Cancer Cell Lines
Source: PLoS One. 2014 Jun 2;9(6):e98330. doi: 10.1371/journal.pone.0098330 (PMC4041891; doi:10.1371/journal.pone.0098330)
Supplement: File S1 — ZIP file containing DMRforPairs output for significant regions. Please start from the html files. (ZIP) [file pone.0098330.s008.zip › figures/32907.pdf]

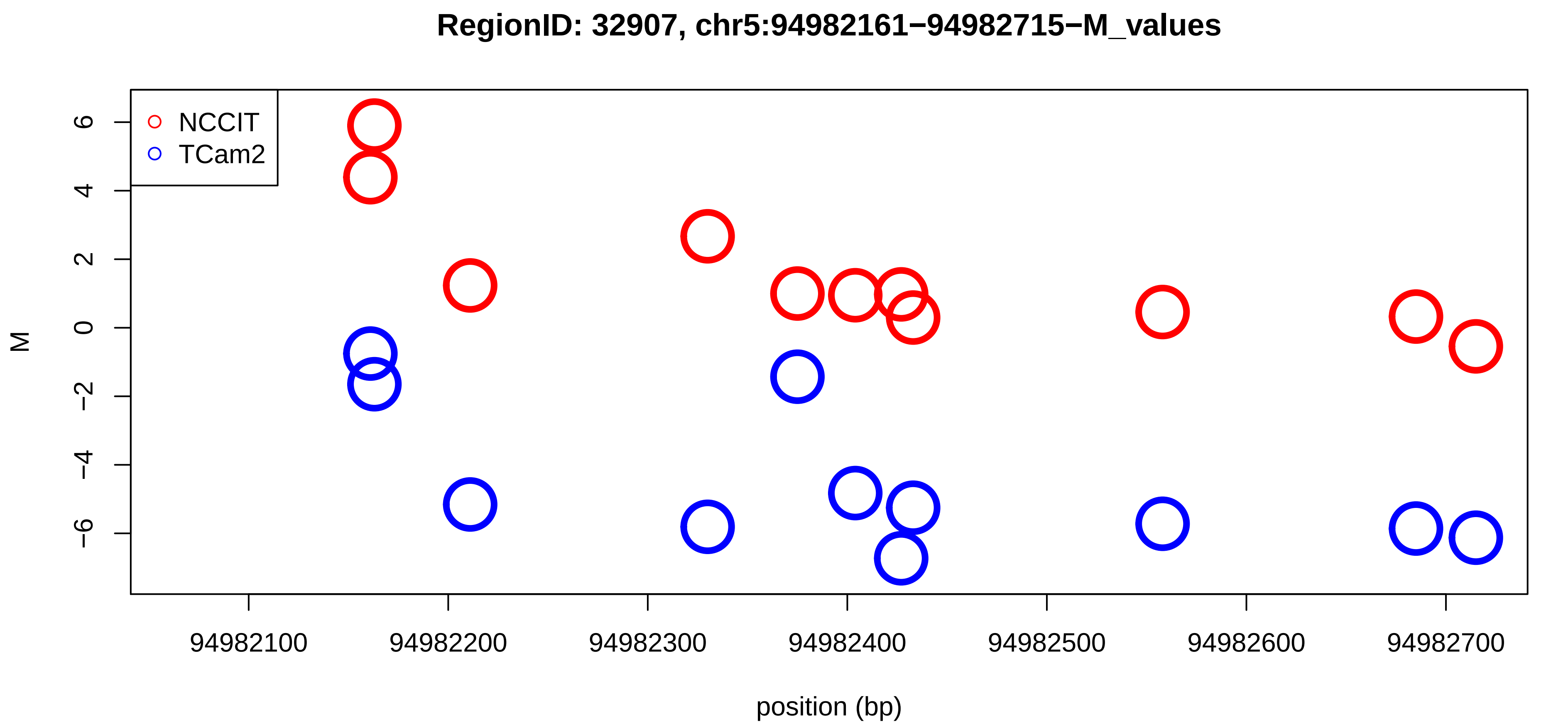
RegionID: 32907, chr5:94982161−94982715−M_values
NCCIT
TCam2
M
6
4
2
0
−2
−4
−6
94982100
94982200
94982300
94982400
94982500
94982600
94982700
position (bp)

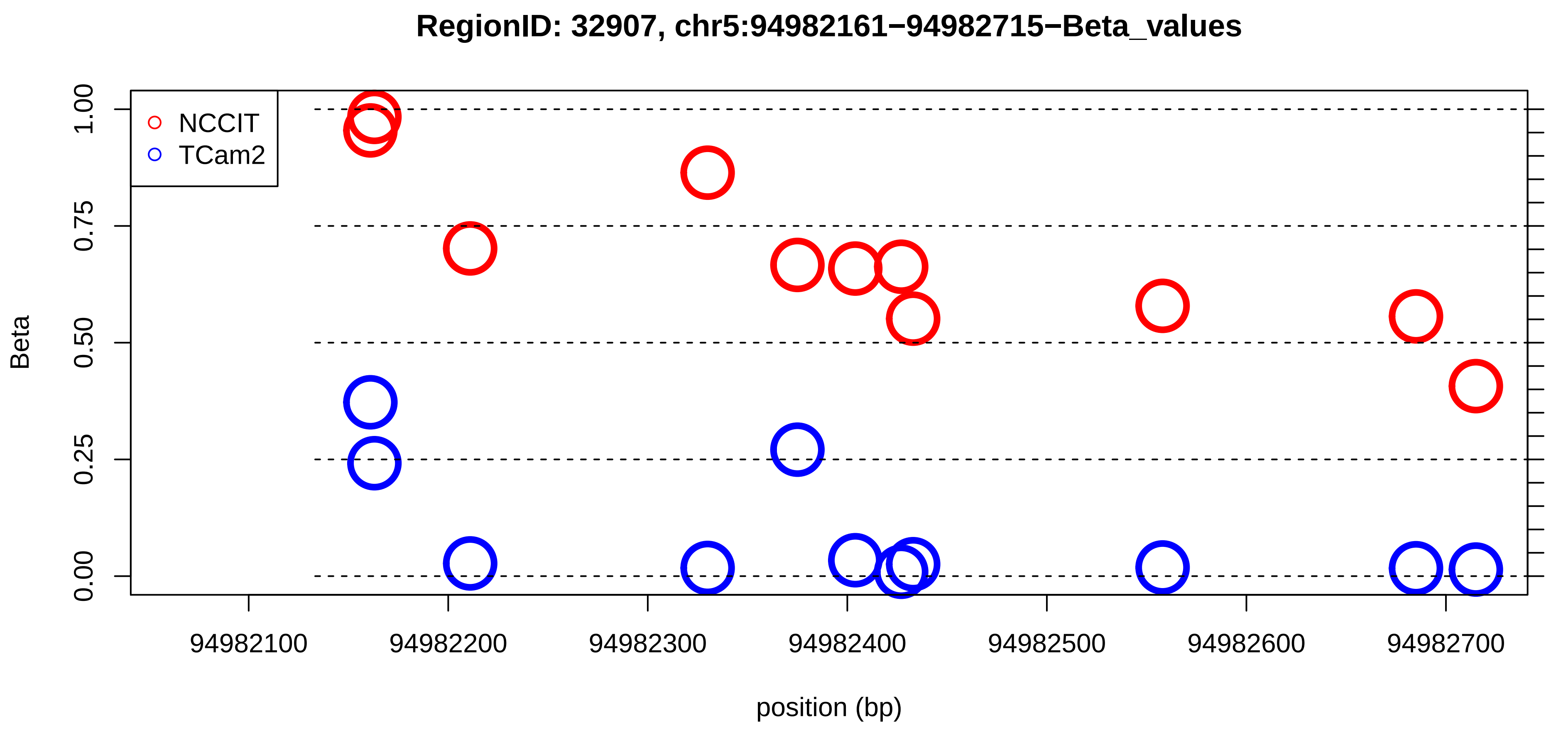
RegionID: 32907, chr5:94982161−94982715−Beta_values
NCCIT
TCam2
Beta
1.00
0.75
0.50
0.25
0.00
94982100
94982200
94982300
94982400
94982500
94982600
94982700
position (bp)

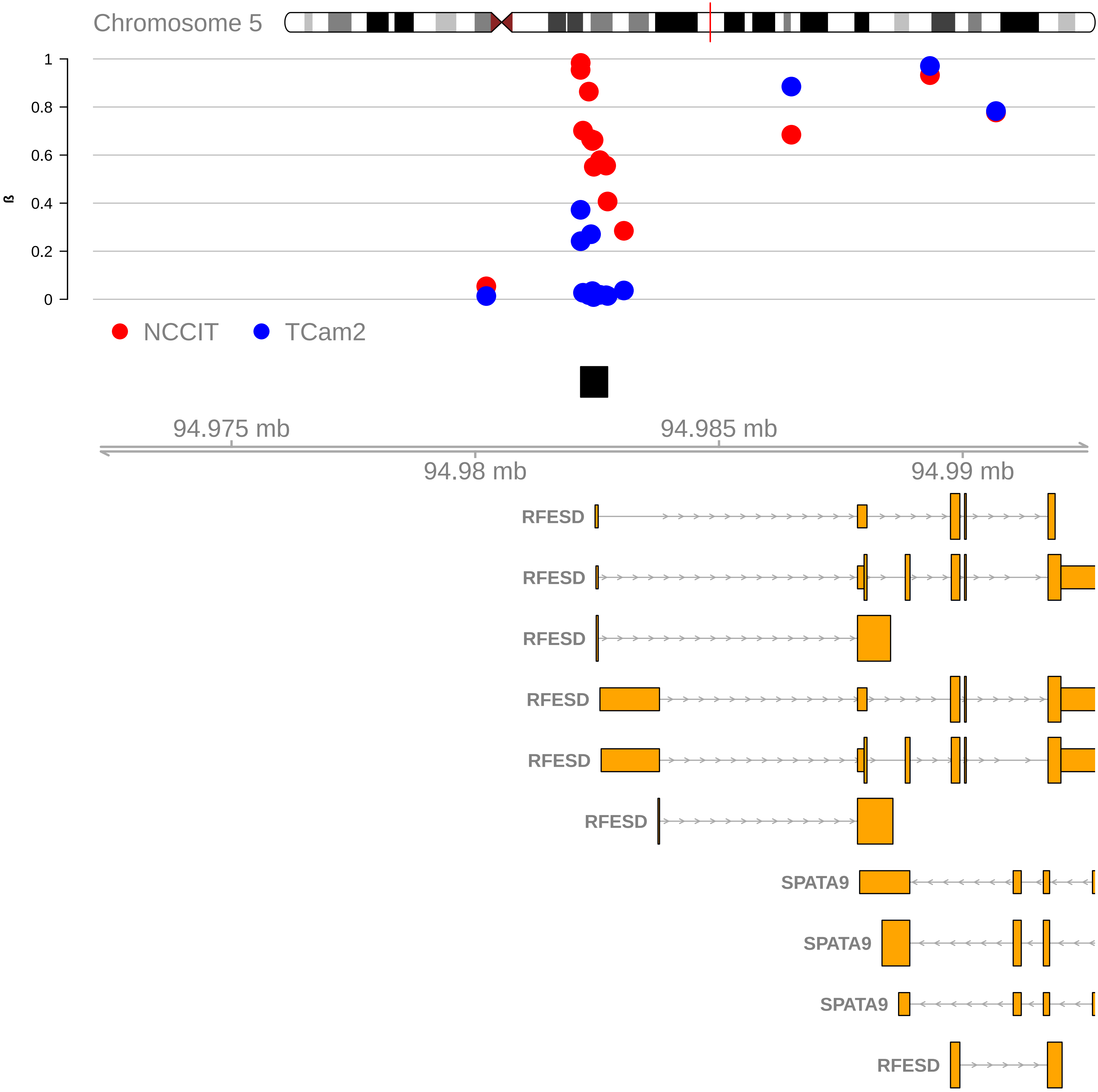

Chromosome 5
1
0.8
0.6
0.4
0.2
0
ß
NCCIT
TCam2
94.975 mb
94.985 mb
94.98 mb
94.99 mb
RFESD
RFESD
RFESD
RFESD
RFESD
RFESD
SPATA9
SPATA9
SPATA9
RFESD
